# Fabric Phase Sorptive Extraction of Selected Steroid Hormone Residues in Commercial Raw Milk Followed by Ultra-High-Performance Liquid Chromatography–Tandem Mass Spectrometry

**DOI:** 10.3390/foods10020343

**Published:** 2021-02-05

**Authors:** Rayco Guedes-Alonso, Zoraida Sosa-Ferrera, José J. Santana-Rodríguez, Abuzar Kabir, Kenneth G. Furton

**Affiliations:** 1 Instituto Universitario de Estudios Ambientales y Recursos Naturales (i-UNAT), Universidad de Las Palmas de Gran Canaria, 35017 Las Palmas de Gran Canaria, Spain; zoraida.sosa@ulpgc.es (Z.S.-F.); josejuan.santana@ulpgc.es (J.J.S.-R.); 2 Department of Chemistry and Biochemistry, International Forensic Research Institute, Florida International University, Miami, FL 33199, USA; akabir@fiu.edu (A.K.); furtonk@fiu.edu (K.G.F.)

**Keywords:** milk samples, steroid hormones, fabric phase sorptive extraction, liquid chromatography, mass spectrometry, fat content, lactose

## Abstract

Hormones in edible matrices, such as milk, are a subject of concern because of their adverse effects on the endocrine system and cell signaling and the consequent disruption of homeostasis in human consumers. Therefore, the assessment of the presence of hormones in milk as potential endocrine-disrupting compounds is warranted. However, the complexity of milk as a sample matrix and the ultra-low concentration of hormones pose significant analytical challenges. Fabric phase sorptive extraction (FPSE) has emerged as a powerful analytical technique for the extraction of emerging pollutants from complex aqueous matrices. FPSE allows for substantially simplified sample handling and short extraction and desorption times, as well as the decreased use of organic solvents. It is considered a green alternative to traditional extraction methodologies. In this work, the FPSE technique was evaluated to perform the simultaneous extraction of 15 steroid hormones from raw milk without employing any sample pretreatment steps. Clean and preconcentrated hormone solutions obtained from FPSE of raw milk were analyzed using ultra-high-performance liquid chromatography–tandem mass spectrometry to achieve low detection limits, which ranged from 0.047 to 1.242 ng·mL^−1^. Because of the presence of many interferents in milk, such as proteins, lipids, and sugar, the effect of fat content on the extraction procedure was also thoroughly studied. Additionally, for the first time, the effect of lactose on the extraction of steroid hormones was evaluated, and the results showed that the extraction efficiencies were enhanced in lactose-free samples. Finally, the optimized methodology was applied to commercial samples of cow and goat milk, and no measurable concentrations of the studied hormones were detected in these samples.

## 1. Introduction

Milk is an essential foodstuff in a balanced diet, and is highly consumed. In fact, milk consumption begins in the very early stages of life, and therefore, its quality must be controlled and verified for commercialization. Some researchers have determined that the residues in milk are chemical substances that may have originated from drugs used in veterinary treatments or from cleaning and other industrial processes in livestock facilities [1]. For this reason, many different organic chemical compounds, such as surfactants, disinfectants, or drugs, could be present in dairy products and affect their quality. European regulations establish the control of some chemical compounds; for example, the Commission Regulation EC 665/2003 establishes the maximum residue limits of veterinary medicinal products in foodstuffs of animal origin [2]. However, to administer these veterinary treatments, it is necessary to carry out a protocol that includes a previous analysis of the animal, the proposed dose of the drug, and the period of administration.

One of the most important groups of veterinary drugs is steroid hormones, which are involved in the regulation of cellular metabolism, development, and physiology, as well as in the metabolism of fat, sugar, or proteins [3,4]. Natural steroid hormones have a very important role in the regulation of cells in animals, and their effects are produced at very low concentrations, in the range of 0.1–1.0 ng·L^−1^. Because they are able to interact and interfere with the endocrine system, steroid hormones, among other compounds, such as pesticides, dioxins, and polychlorobiphenyls, are considered endocrine active substances [5]. As veterinary drugs, natural and synthetic steroid hormones are extensively used because of their therapeutic properties [6]. This group of emerging pollutants can be administered to animals for three main purposes: to treat certain illnesses, to address reproductive disorders, and to improve growth rates of animals [7]. In fact, the Council Directive 96/22/EC of the European Commission [8] states that Member States may authorize “the administering to farm animals, for therapeutic purposes, of estradiol 17β, testosterone and progesterone and derivatives which readily yield the parent compound on hydrolysis after absorption at the site of application.” However, the misuse of steroid hormones in the cattle industry may lead to the presence of these compounds in food. Some examples of this misuse are the administration of higher doses than acceptable, inappropriate injection sites, failure to remove injections or implants, or the usage of illegal substances [9]. Despite the controversy about the use of steroid hormones in cattle farming, some countries, such as the US, permit the use of estrogens or anabolic substances in the management of animal reproductive systems, although the use of steroid hormones as growth promoters has decreased in recent years in North America [10].

The presence of steroid hormones in milk has been reported by different studies, and the concentrations are in the range of ng·L^−1^ [5,11], but the bioavailability of orally consumed hormones is low. Generally, only 5% of administered hormones are available in the circulation, and moreover, the percentage of free hormones in plasma is a low fraction of bioavailable hormones [12]. Thus, plasma hormone levels are significantly lower than the concentrations found in milk [11]. Nevertheless, the presence of hormones in edible matrices is a concerning issue, because they interfere with the homeostasis of human steroid hormones. In fact, some studies have linked the intake of milk from pregnant cows with some changes in the levels of human estrogens and androgens, or in the mobility and morphology of sperm [13,14]. Usually, steroid hormones are present in milk as conjugated forms that are not biologically active, but in the human gut, they can be transformed into their free forms [15]. Although they are naturally occurring compounds, high serum estrogen concentrations have been associated with certain cancers, such as breast, uterine, and ovarian cancers in women [16] and testicular or prostate cancer in men [17].

To satisfactorily measure steroid hormones at low levels in milk samples, it is necessary to develop analytical procedures that permit their extraction and preconcentration. Additionally, sample preparation steps before extraction procedures are critical in the successful analysis of biological samples [18]. The analysis of milk, as well as other foodstuffs, can be problematic, because it contains many compounds, including lipids, proteins, carbohydrates, fats, and minerals, that may interfere with the determination of target compounds. One example is phospholipids, which possess amphiphilic properties [19] that can produce undesirable interactions and, consequently, a low recovery efficiency. According to the available literature, steroid hormones are commonly isolated using solid-phase extraction [20]. However, pretreatment steps are frequently necessary to prepare the sample for extraction. Common pretreatments include pH adjustment and enzymatic digestion/hydrolysis prior to the extraction process [11,17,21,22], as well as other sample preparation steps, such as centrifugation [21,22], derivatization [17], or liquid–liquid extraction [11]. Some of these steps are also used in novel extraction techniquees, such as magnetic solid-phase extraction, developed by Ding et al. [23]. The main problem associated with the necessity of performing sample pretreatments is that they can lead to substantial losses in the overall recovery of the extraction and, therefore, lead to poor detection limits [24].

For all of the above reasons, in recent years, several new extraction techniques have been developed that require minimum sample preparation, as well as low amounts of the sample. For example, fabric phase sorptive extraction (FPSE), developed by Kabir and Furton in 2014, is a novel and green sample preparation technique that uses a natural or synthetic fabric substrate that has been chemically coated with a sol–gel sorbent, resulting in a sensitive, portable, and easy-to-handle microextraction device [25]. One of the main advantages of FPSE is that it can be used without modification of the sample, allowing for the direct extraction of analytes. The use of fabric phase sorptive extraction has been previously reported in several works for the extraction of emerging pollutants, such as sulfonamides [26], amphenicols [27], penicillin antibiotics [28], bisphenol-A, and other endocrine-disrupting compounds (EDCs) [29] in raw or intact milk with minimum sample pretreatment. The lipophilic behavior of most steroid hormones makes them promising candidates for extraction by FPSE. Moreover, it is necessary to use selective and sensitive detection techniques because of the trace concentrations of steroid hormones in milk. In this regard, liquid chromatography–tandem mass spectrometry (LC–MS/MS) methods have been recognized as effective analytical techniques that afford the specificity and detection limits necessary for the measurement of steroids in both environmental and biological samples [30,31,32,33].

In this work, the extraction efficiencies of three different sorbent materials used in fabric phase sorptive extraction devices were studied in order to identify the best sorbent chemistry to extract 15 steroid hormones from raw milk. The extraction conditions were optimized following a factorial experimental design, and, after that, all of the analytical parameters of the extraction procedure with the FPSE membrane that showed better extraction efficiencies were studied. The FPSE–UHPLC–MS/MS method was applied for the first time to commercial milk samples with different characteristics to evaluate the presence of 15 hormones from four different families of steroid hormones: estrogens, androgens, progestogens, and glucocorticoids (Table 1).

## 2. Materials and Methods

### 2.1. Solvents, Reagents, and Standard Preparation

The steroid hormones under study were all purchased from Sigma-Aldrich (Madrid, Spain), and all of the standards used were over 99.0% purity. Stock solutions of 1000 mg·L^−1^ were prepared by dissolving the appropriate amount of the compound in methanol, and then they were stored in amber vials at −20 °C. The mixture stock solution was prepared by diluting primary stock solutions to a concentration of 10 mg·L^−1^ in methanol. Working solutions were prepared daily from the mixture stock solution. Additionally, three internal standards were used to mitigate the signal changes produced by matrix effects in the analysis of commercial samples. Estrone D2 (E1–D2) was used as the internal standard (IS) for estrogens, testosterone D3 (TES–D3) was used in the determination of androgens, and progesterone D9 (PRO–D9) was used as the IS in the determination of progestogens and glucocorticoids. The concentration of the internal standards used was 100 µg·L^−1^. HPLC-grade methanol used in the FPSE procedure was obtained from Panreac Química (Barcelona, Spain), while the water used in the extraction and in the cleaning step of FPSE devices was provided by a Milli-Q system (Millipore, Bedford, MA, USA). LC–MS-grade water and methanol (used as the mobile phase) were both from Panreac Química (Barcelona, Spain), as was the ammonia used to adjust the pH of the mobile phase.

### 2.2. Sol–Gel Sorbent Coated FPSE Media

Three different FPSE membranes were evaluated in this study: membranes coated with sol–gel Carbowax^®^ 20M (sol–gel CW20M), sol–gel poly(tetrahydrofuran) (sol–gel PTHF), and sol–gel poly(ethylene glycol 300) (sol–gel PEG300). It should be noted that, in both the sol–gel CW20M and sol–gel PEG300 sorbent coatings, poly(ethylene glycol) polymers were used as organic polymers with different average molecular weights (20,000 and 300 Da, respectively). All FPSE membranes measured 20 × 25 mm and, consequently, provided an extraction surface area of 500 mm^2^ on each side.

### 2.3. Instrumentation

To perform the separation and determination of target steroid hormones, an ultra-high-performance liquid chromatography system coupled to a triple quadrupole detector (UHPLC–MS/MS) was used. The system consists of an ACQUITY Quaternary Solvent Manager (QSM), which is used to load samples and to wash and equilibrate the analytical column, as well as an autosampler capable of injecting up to 21 samples equipped with an injection syringe of 25 μL, a column oven, and a triple quadrupole detector (TQD), which were all from Waters (Barcelona, Spain). The detection parameters for steroid hormones were optimized in a previous work [31], and are summarized in Table S1. To carry out the separation of the target steroid hormones, an ACQUITY UHPLC BEH Waters C18 (50 mm × 2.1 mm, 1.7 μm) analytical column from Waters (Barcelona, Spain) was used. The injected sample volume was 10 µL, and the analyte separation was carried out using water with 0.1% (*v*/*v*) ammonia and methanol without additives at a flow rate of 0.3 mL∙min^−1^ in gradient mode for 6.5 min [31].

### 2.4. Sample Collection and Preparation

Milk samples were purchased from food stores in Gran Canaria (Spain). Different types of commercial milk were analyzed to evaluate the effect of lipids or lactose on the extraction procedure. For this analysis, skimmed (0% fat), full-fat milk (3.5% fat), and lactose-free skimmed cow milk were analyzed. Commercial semi-skimmed (1.5% fat) goat milk was also analyzed. The samples were stored at room temperature prior to analysis and, once opened, were kept at 4 °C. In all cases, the samples were analyzed before the expiration date. Because of the advantageous characteristics of the fabric phase sorptive extraction technique, no preparation steps were necessary for milk samples. In the experiments that required the sample to be spiked, the appropriate amount of working standard solution was added to the sample before the extraction procedure, aiming to minimize the amount of organic solvent added as much as possible.

### 2.5. Statistical Analysis

Fabric phase sorptive extraction involves several variables that must be studied, such as sample and elution solvent volumes and extraction times. Because of the possible interactions among these variables, a factorial experimental design was used. This technique permits the reduction of the required number of experiments, thus limiting reagent use and experimental times and, as Tarley et al. stated, allowing free interaction with data, the making of comparisons, and the seeking of familiarities [34]. The combination of factorial designs and the study or partial correlations of the involved variables permit the establishing of the most suitable combination of the tested parameters of the variables that affect the extraction procedure. For these reasons, factorial designs are frequently used as a chemometric tool in biological, environmental, and pharmaceutical studies of food applications and analytical method development [35]. In this study, the statistical analysis was conducted using Minitab 17 Statistical Software (Coventry, UK). The software permits the modeling of the factorial design, as well as the study of the effects of variables on the extraction procedure and the interactions among variables.

### 2.6. FPSE Procedure and Target Hormone Quantification

To activate the sol–gel sorbent that coated the FPSE device, fabric membranes were immersed in 2 mL of a mixture of methanol/acetonitrile (50:50, *v*/*v*) for 5 min, and were then submerged in 2 mL of ultrapure Milli-Q water for another 5 min. Subsequently, the appropriate volume of milk was placed in glass vials with a Teflon-coated magnetic stirrer, and the fabric membrane was submerged in the sample solution and stirred at 700 rpm for the optimum extraction time. As an important step, contact between the fabric device and the sample was confirmed during the extraction. After that, the fabric device was removed from the vial and submerged in the appropriate volume of eluent solvent to perform the elution of the analytes. The obtained extracts were then centrifuged at 4000 rpm for 10 min and filtered through 0.22 µm membrane syringe filters in order to eliminate possible interferents. The optimized method permits the whole extraction of the target steroid hormones from milk in 35 min, which is a shorter time than other extraction techniques, because in those it is necessary to perform different pretreatments as well as washing steps. To avoid potential carryover effects, the devices were washed by immersing them in 2 mL of water for 5 min and then in 2 mL of methanol for an additional 5 min. Finally, the fabrics were dried for 10 min before storage. To determine the optimum FPSE sol–gel device for the quantification of steroid hormones, absolute recoveries were calculated by comparing the signal after the FPSE procedure with that of a standard prepared in methanol. For the optimization of FPSE with the optimum sol–gel device, relative recoveries were calculated by comparing the chromatographic areas of an FPSE extract spiked with target analytes with the chromatographic areas obtained from the extract of a spiked sample, as shown in Equation (1).

(1) $$\mathrm{Recovery}\;\left(\%\right)=\frac{{\left(\mathrm{Peak}\;\mathrm{area}\right)}_{\mathrm{spiked}\;\mathrm{sample}}}{{\left(\mathrm{Peak}\;\mathrm{area}\right)}_{\mathrm{spiked}\;\mathrm{extract}}}\times 100$$

## 3. Results and Discussion

### 3.1. Selection of Fabric Phase Sorptive Extraction Media

In contrast to sorbents used in other microextraction techniques, fabric phase sorbent extraction leverages the hydrophilic/hydrophobic properties of the substrate to supplement the polarity of the extraction sorbent [26]. The polarity of target steroid hormones varies from medium to low, with logarithmic values of the octanol–water partition coefficient (log K_ow_) ranging from 1.46 to 5.07. For this analysis, a balance between membranes with hydrophilic and hydrophobic properties and a cellulose substrate as a fabric support of the sol–gel sorbent coatings were used. As sorbent coatings, three different sol–gel sorbent materials were studied. Two were based on poly(ethylene glycol)—Carbowax^®^ 20M (CW–20M) and PEG 300—and the other was based on poly(tetrahydrofuran) (PTHF). All of them had medium polarity (as the organic polymer is one of several building blocks of the sol–gel sorbent that collectively determine the overall polarity of the composite material), which is appropriate for extracting the target steroid hormones. One of the main differences among sorbents was their loadings. Carbowax^®^ 20M devices had a loading of 43.2 mg, while PEG 300 and PTHF had loadings of 20.5 and 19.8 mg, respectively. It is worth mentioning that the amount of sorbent loading determines the maximum amount of analyte(s) that an FPSE membrane can extract under equilibrium extraction conditions. For trace- and ultra-trace-level concentrations of the target analyte(s), FPSE membranes never reach their saturation points. Owing to the large number of available FPSE sorbent chemistries, the developers have created an extraction recovery model for each FPSE sorbent chemistry, which allows researchers to predict the extraction recovery of an organic compound using its log K_ow_ value. The validity of the extraction recovery models has been reported in several studies [26,36,37]. These extraction recovery models were created using analyte solutions in deionized water. As a result, moderate deviation is expected when a sample matrix, such as milk, contains excessive interferents. The three FPSE devices were chosen according to the expected recoveries for the target steroid hormones. The expected recoveries were calculated from the model equations (Table S2). For sol–gel Carbowax^®^ 20M, the theoretical recoveries for the 15 steroid hormones studied ranged between 32.51% and 102.95%, with an average extraction efficiency of 70.55%. Similar theoretical recoveries were predicted for sol–gel PEG 300 and PTHF, which ranged from 29.51% to 71.15% and from 28.73% to 74.31%, respectively. The average extraction efficiencies for these two sorbents were predicted to be 58.61% for sol–gel PEG 300 and 60.51% for sol–gel PTHF.

### 3.2. Optimization of FPSE Conditions

Fabric phase sorptive extraction involves several key variables that can affect the extraction procedure, therefore, they must be optimized in order to achieve higher extraction efficiencies. These variables include sample and eluent solvent volumes and extraction and elution times. Methanol was used as the solvent extractant in accordance with the protocol provided by FPSE developers and the results of our previous study on the extraction of steroid hormones [31]. In this study, a 2^4^ full factorial experimental design was performed, in which the four variables were studied at two levels (low and high values are denoted by − and +, respectively). The FPSE conditions were studied for each fabric membrane. The low and high values of each variable are shown in Table 2, and were chosen on the basis of values in the standard protocol for FPSE, as well as the characteristics of the milk samples. To prevent large quantities of interferents in milk, sample volumes were less than 5 mL, and the maximum extraction and elution times were 30 and 10 min, respectively, to avoid long analysis times. The extractant volumes were 1 and 2 mL to prevent overdiluting the extracted analytes. Notably, only 1 mL of desorption solvent was necessary to completely submerge the FPSE membrane.

#### 3.2.1. Factorial Experimental Designs for Selected FPSE Media

First, the different parameters that affect FPSE were optimized for each FPSE membrane in order to obtain the maximum extraction efficiency with each fabric coating. To perform the factorial experimental design, the different experiments were randomized in order to minimize possible carryover effects between analyses. Table 3 shows the values used for each experiment. Milk samples were spiked before FPSE to achieve a final concentration of 200 ng·mL^−1^ in the extract.

**Figure 1:**
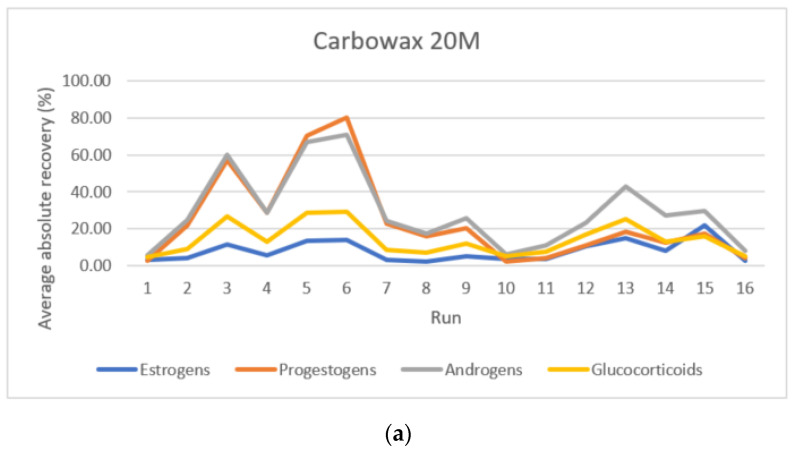

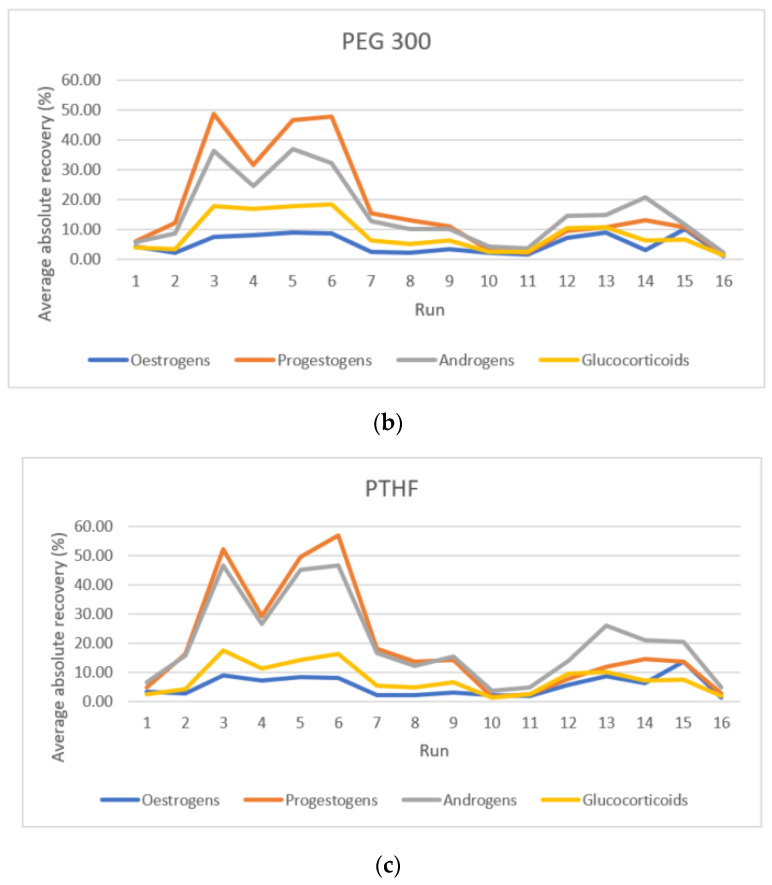
Average absolute recoveries for analytes of the different steroid hormone families under study for the different runs of the factorial experimental design. (**a**) Carbowax^®^ 20M, (**b**) PEG 300, and (**c**) PTHF.

Figure 1 shows the extracted concentrations of each steroid hormone under study for the different runs performed for sol–gel Carbowax^®^ 20M (Figure 1a), sol–gel PEG 300 (Figure 1b), and sol–gel PTHF (Figure 1c). Runs 3, 5, and 6 produced the highest extracted concentrations for the three FPSE devices under study, while the rest of the runs followed similar trends for all of the extracting media. However, for the runs with the most effective extraction, the efficiencies of sol–gel PEG 300 and sol–gel PTHF were lower than that of sol–gel Carbowax^®^ 20M. Specifically, for sol–gel CW20M, the extract contained up to 1400 µg·L^−1^ of spiked hormones, while for sol–gel PEG 300 and sol–gel PTHF, the sum of the extracted concentrations was between 800 and 900 µg·L^−1^. Important differences were also observed among extraction efficiencies for the different families of steroid hormones. In Figure 1, the amounts of extracted progestogens and androgens (orange and grey bars) are significantly higher than those of glucocorticoids and estrogens (yellow and blue bars). In particular, for progestogens and androgens, the maxima of combined extraction efficiencies were 81.8% and 72.1%, respectively, while those for estrogens and glucocorticoids were 21.7% and 29.2%, respectively. In light of these results, sol–gel CW20M was chosen as the optimal extraction membrane, because it resulted in the best extraction efficiencies for the four families of steroid hormones under study.

#### 3.2.2. Interactions of Variables in Selected FPSE Membranes

**Figure 2:**
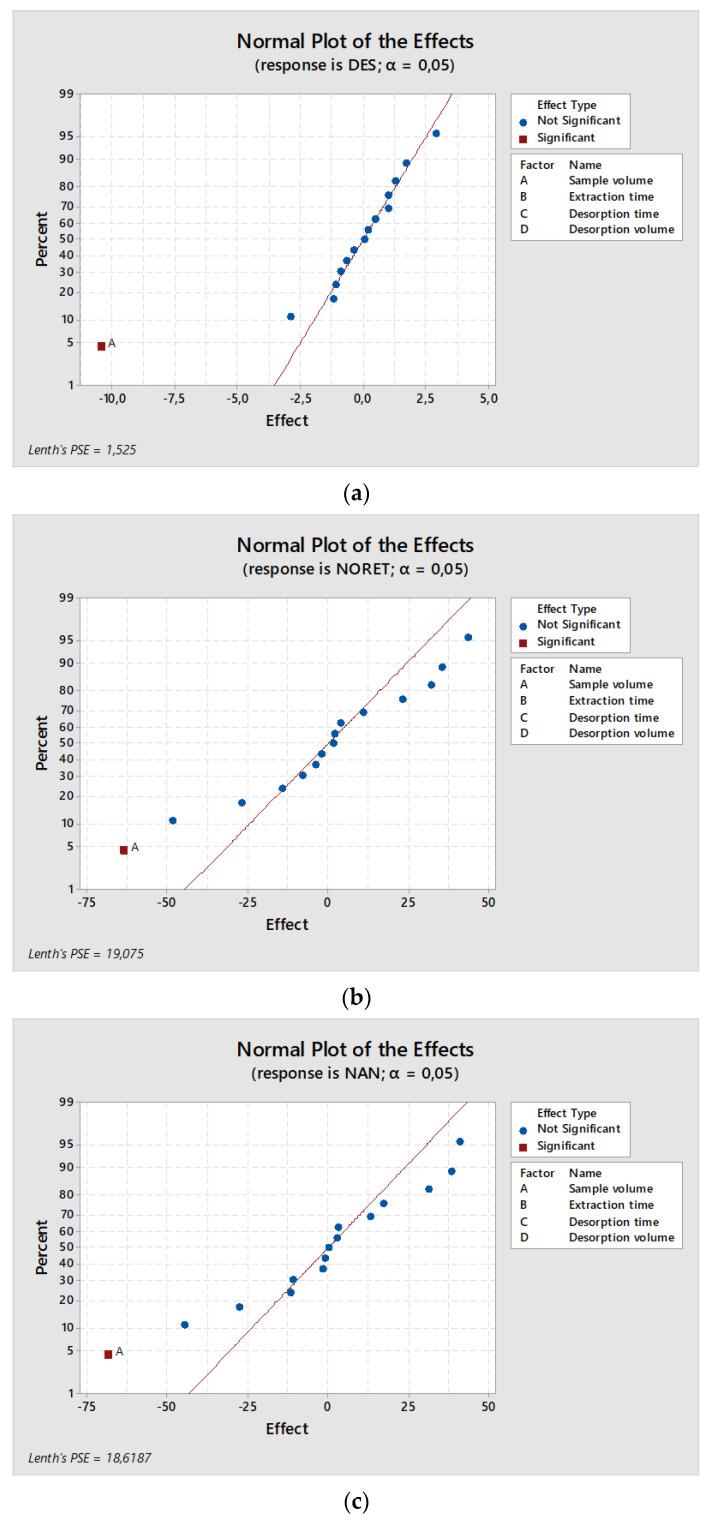

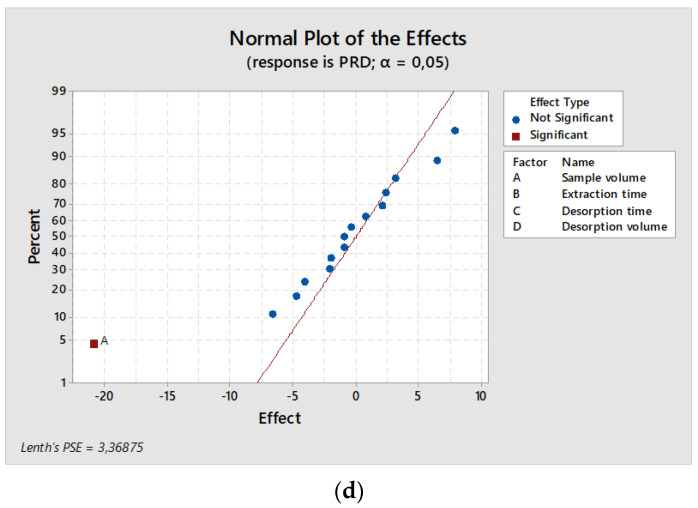
Normal plots of the effects for four steroid hormones, one from each family, under study: (**a**) estrogens, (**b**) progestogens, (**c**) androgens, and (**d**) glucocorticoids.

After choosing the optimum FPSE membrane, the variables that affect FPSE and the interactions between them were studied in order to determine the best combination values of the variables and their effects on the extraction process. For this purpose, Pareto charts and normal plots of the effects were built for each compound to evaluate the effect of each variable and the relationships among them (Figure S1). Figure 2 shows the normal plots of the effects of several compounds under study. In most cases, the normal plots are similar to those of diethylstilbestrol (DES), norethisterone (NORET), nandrolone (NAN), and prednisone (PRD), which show that the sample volume is the only significant variable among those studied. This behavior is apparent in all estrogens and glucocorticoids, the two families with lower extraction efficiencies, except for ethynylestradiol (EE) and cortisone (COR), which also reflect significant effects for the combination of desorption time and volume (for EE) and sample volume and desorption time (for COR).

For the families with higher extraction efficiencies, progestogens and androgens, the normal plots were more variable, and only NORET and NAN revealed a significant variable (sample volume). For the rest of the compounds of these two families, other combinations of variables were also significant, but, in all cases, the sample volume (variable A) was included, which indicated the high importance of sample volume in the extraction process. This can be explained by the interferents present in milk, which can produce notable changes in the extraction efficiency of the technique.

After evaluating the effects of the different variables, it was necessary to establish their optimum values; therefore, partial correlations between each variable and steroid hormone were calculated, and the results are summarized in Table S3. The calculation of these correlations confirmed the results obtained in the Pareto charts and normal plots of the effects: that is, the sample volume was the only significant variable in the extraction process. Specifically, all of the compounds had negative Pearson correlation values and *p*-values below 0.05 (except for progesterone (PRO), *p*-value = 0.06). The negative correlation between the sample volume and extraction efficiency was more pronounced for estrogens and glucocorticoids (as the normal plots show), with Pearson correlations between −0.730 and −0.893, which indicated that higher volumes of the sample led to a loss in the extraction efficiency of these compounds. A similar trend was observed for the three compounds of the androgen family. The Pearson correlation values ranged between −0.615 and −0.850, which also indicated a negative correlation between the sample volume and extraction efficiency. Finally, for progestogens, the correlations were not as high, and were between −0.480 and −0.574. These negative correlations reflected the first hypothesis about the effect of interferents on the extraction efficiency, because the higher the volume of milk used for extraction, the lower the hormone extraction efficiency. This effect could be related to the capacity of steroid hormones to bind to proteins, which affects the interaction between the steroid and the extraction device. This problem has been stated for other compounds, such as tetracycline antibiotics [18]. Given this trend, 1 mL of the sample was chosen as the optimum value. No lower volumes were evaluated, because 1 mL is the minimum necessary to completely submerge the fabric media in the sample. The other three studied variables did not have notable correlations with the extraction efficiency, and the Pearson correlation values for these three variables were from −0.158 to 0.217. This absence of correlation permitted the minimization of the extraction and elution times, which were fixed at 10 and 5 min, respectively. Additionally, to minimize the dilution of the samples, 1 mL of methanol as the extractant solvent was chosen as the optimum value. This small volume contributed to enhancing the sensitivity of the method, as well as reducing the time of the evaporation step.

### 3.3. Analytical Parameters

The evaluation of the variables that affect the FPSE process was validated by studying the linearity, reproducibility, and sensitivity of the technique, as well as the recoveries of the extraction. Table 4 includes the validation parameters.

Linearity was determined by constructing calibration curves for each compound under study. The linear range of the calibration was between 1 and 400 ng·mL^−1^, and the concentration of the internal standards was 100 ng·mL^−1^. Each point of the calibration curve was produced from a standard prepared in methanol by diluting a stock solution that contained a mixture of the 15 steroid hormones under study. To determine the absolute recoveries of the tested FPSE media, external calibration curves were used, while internal calibration curves were built for the determination of target steroid hormones in commercial samples. In all cases, linear regression coefficients (r^2^) over 0.992 were achieved.

The expected concentrations of steroid hormones in milk samples are low, and for this reason, it is necessary to develop an analytical methodology with low detection limits that allows these trace contaminants to be determined. For the determination of method detection and quantification limits, the signal-to-noise ratio was used. The detection limits (MDLs) were calculated as the concentrations that produce a signal-to-noise ratio equal to 3, and the quantification limits (MQLs) were the concentrations that resulted in a signal-to-noise ratio equal to 10. Signal-to-noise ratios were calculated from a spiked sample of skimmed milk. As shown in Table 4, low MDLs between 0.012 and 1.242 ng·mL^−1^ were achieved, which indicated that the extraction and detection procedure was capable of determining up to nanograms of steroid hormones in one milliliter of milk.

**Figure 3:**
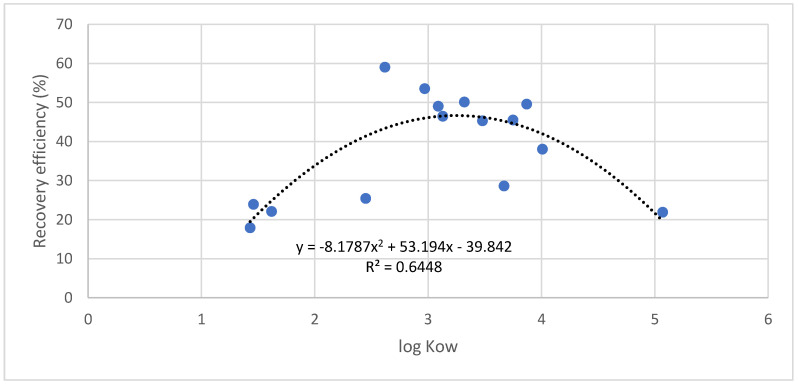
Relationship between experimental recovery efficiencies and the octanol–water partition coefficient.

Regarding the recoveries of the extraction methodology, it is important to highlight that the extraction of the analytes using FPSE, as in other microextraction techniques, is not exhaustive. During FPSE, an equilibrium is established between the extracting membrane and the sample after the optimum extraction time. Furthermore, matrix interferents can affect the extraction process. For these reasons, it is necessary to evaluate the extraction efficiency of the developed methodology. Table 4 summarizes the extraction recoveries achieved for skimmed milk at a concentration of 100 ng·mL^−1^. The octanol–water partition coefficient was compared with the experimental recoveries in order to establish a model of the extraction of compounds from milk samples using sol–gel Carbowax^®^ 20M media. As shown in Figure 3, the recoveries fit an order-2 polynomial curve, with a maximum between log K_OW_ values of 3 and 3.5, and minimum recoveries for compounds, with a log K_OW_ lower than 2 as glucocorticoids or higher than 3.5 as some estrogens. The fit of the linear regression was also significant (r^2^ = 0.503) when data from diethylstilbestrol and 17α-ethynylestradiol (log K_OW_ = 5.07 and 3.67, respectively) were removed from the study in the range of log K_OW_ from 1.5 to 3.5. This indicated that the developed FPSE method was especially appropriate for androgens and progestogens, which showed log K_OW_ between 2.6 and 3.9. For these compounds, the extraction recoveries were in the range of 45 to 59%. The loss in the extraction efficiency for compounds with high log K_OW_ was predicted in the models provided by the FPSE media developers and confirmed in this study; the only observed difference was that the loss in the extraction efficiency was more accentuated in the model built with the results of steroid hormones. In general, the less polar compounds were observed to have better recoveries than the most polar ones.

The signal changes that were produced by the matrix effects were evaluated by comparing a spiked extract after the FPSE procedure with a standard prepared in methanol. The evaluation of the matrix effect is a key factor in the development of analytical procedures when mass spectrometry detection is used, especially with complex matrices, such as milk. Because of the multiple interferents extracted from milk during FPSE, the signals of most of the compounds were suppressed by between 29.3 and 86.9%. For this reason, three internal standards were used to mitigate the signal suppression produced by the matrix. The election of these three internal standards was performed considering the molecular structures of the different target steroids and their subsequent detection behavior. In this regard, for estrogens, a deuterated C-18 steroid hormone was chosen (estrone-d2). For androgens, the IS chosen was a C-19 steroid hormone, specifically the major androgen, testosterone-d3. Finally, progesterone-d9 was chosen as the IS for target steroid hormones with carbon structures of 21 C, such as progestogens and glucocorticoids. The election of major hormones as representative internal standards for their respective families has been widely used as a simplified strategy to overcome matrix effect problems by many authors [38,39]. The different internal standards used for the different families of steroid hormones under study are specified in Table 4.

### 3.4. Effect of Lipids and Lactose on Extraction Efficiency

The presence of some components in milk, such as proteins or fat, may result in several problems in the extraction process of pollutants. For this reason, some pretreatment processes, such as protein precipitation and defatting, are necessary prior to the extraction of emerging pollutants from milk samples [29]. In this regard, Samanidou et al. evaluated the effects of fat on the extraction efficiency of FPSE media, and concluded that the higher the fat content in milk, the greater the loss in the extraction efficiency [28]. A similar observation was reported by R. Mesa et al. [29]. However, when FPSE is used for the extraction of pollutant residues, such as antibiotics or endocrine-disrupting compounds from milk, the deproteinization process results in a substantial loss in the extraction efficiency, because a proportion of the compounds present in milk is eliminated with the proteins and fats during this pretreatment process. For these reasons, a study on the effect of the fat content of milk was performed. Additionally, the potential effect of lactose on the extraction efficiency was studied.

**Figure 4:**
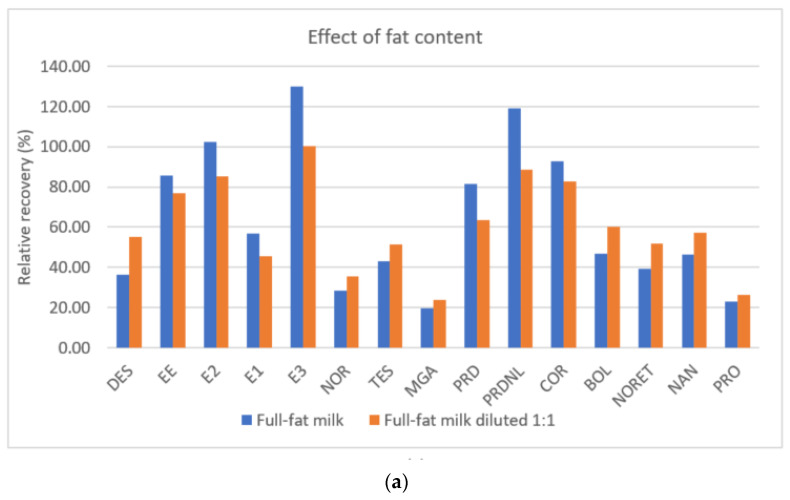

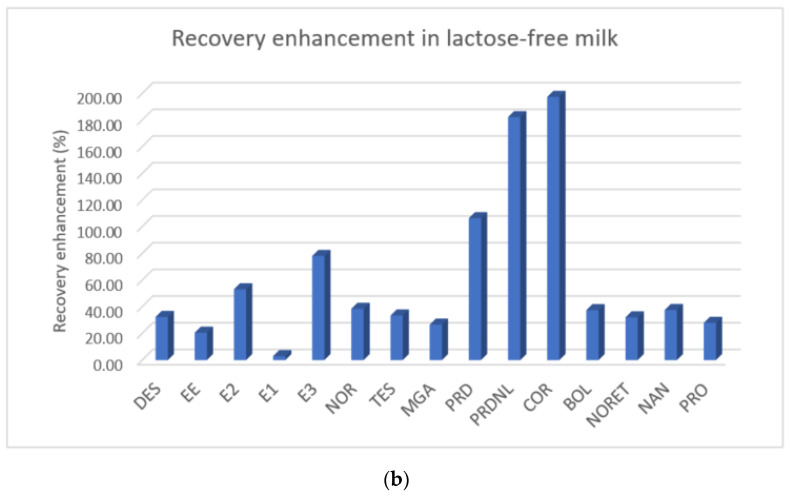
Effects of fat content (**a**) and lactose (**b**) on the extraction efficiency of the optimized FPSE method. Comparisons made with average recoveries (*n* = 3).

Figure 4a shows the relative recoveries obtained in full-fat milk (3.5% fat) and full-fat milk diluted 1:1 with ultrapure water, and Figure 4b shows the relative enhancements obtained for lactose-free skimmed milk. As illustrated in Figure 4a, for most of the target hormones, the higher the content of fat in the milk, the higher the loss in the removal efficiency, as has been reported in other studies that used FPSE in milk samples for other emerging pollutants. The losses are not as significant in compounds with low extraction recoveries (glucocorticoids and estrogens), for which the relative recoveries are between 80 and 120% (except for diethylstilbestrol and estrone). However, for the families of steroid hormones with better recoveries (androgens and progestogens), the presence of fat reduces the extraction recovery by an average of 65%. When full-fat milk is diluted with ultrapure water, the extraction efficiencies of estrogens and glucocorticoids slightly decrease (between 10 and 20%), while the losses in extraction efficiencies for androgens and progestogens are not as pronounced (average loss of 55%). Because of these results, diluting the milk sample in order to reduce the concentration of fat content was not performed, also because the extraction efficiency is not significantly improved, and it simultaneously dilutes the target steroid hormones present in the milk sample.

This study is the first to evaluate the effect of lactose on the extraction of steroid hormones from milk samples by analyzing samples without lactose. For this analysis, the recoveries obtained from lactose-free skimmed milk were compared with those from a sample of skimmed milk. The results show that, in the lactose-free milk analysis, the extraction efficiency is enhanced in the group of glucocorticoids, which increases from recoveries of 17–23% to 49–65%, translating to an enhancement of extraction efficiency between 100 and 200%. For estrogens, androgens, and progestogens, an enhanced extraction efficiency is also observed, but it is not as significant compared with glucocorticoids. Extraction efficiencies of most of the compounds are enhanced by between 25 and 50%, except for estrone (2.9%), 17α–ethynylestradiol (20.5%), and estriol (78.1%). This suggests that lactose produces some effects on the extraction procedure, and thus, this compound may be considered an interferent in the analysis of organic compounds in milk.

### 3.5. Application of the Optimized Methodology to Commercial Samples

**Figure 5:**
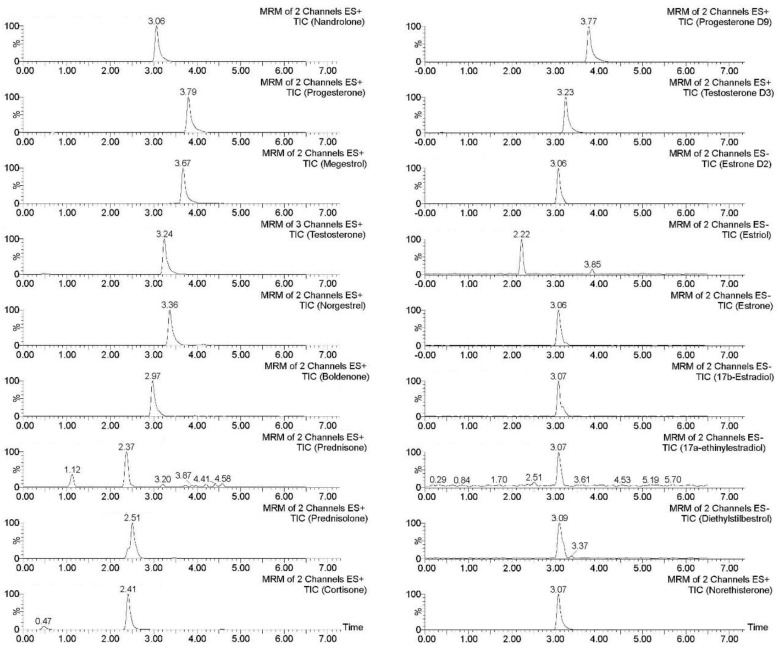
Chromatogram of a skimmed milk sample spiked with 100 ng·mL^−1^ of each of the selected hormones obtained after the application of the optimized FPSE–UHPLC–MS/MS methodology.

The proposed methodology was used for the analysis of milk samples from three different brands to evaluate the presence of target hormones in commercial samples. These samples included skimmed milk (<0.5% fat), lactose-free skimmed milk, full-fat milk (3.5% fat), and semi-skimmed goat milk (1.5% fat), and each milk sample was analyzed in triplicate. No hormones were detected in any sample under study. For this reason, to demonstrate the applicability of the method, one of the samples of skimmed milk was spiked with all of the selected hormones at a concentration of 200 ng·mL^−1^ after verifying that it did not have any steroid hormones above the limit of detection. The chromatogram of the spiked sample in Figure 5 is very similar to those obtained using exhaustive extraction methods, such as SPE or liquid–liquid extraction [3], which means that the developed method presents very good selectivity. In comparison with the study conducted by Malekinejad et al., good selectivity is obtained for all target steroid hormones, including estriol, which showed unsatisfactory selectivity in that study [17]. Considering that some authors have determined the presence of naturally occurring estrogens and androgens in milk at concentration levels in the range of 0.010–0.100 ng·mL^−1^ [11,40], this methodology could be adopted to evaluate the presence of steroid hormones in contaminated milk that could be considered a risk to the health and well-being of the consumers.

## 4. Conclusions

A novel FPSE–UHPLC–MS/MS method was developed for the simultaneous extraction and determination of 15 steroid hormones from four different types of raw milk. From a large number of available FPSE membranes with unique selectivity, three membranes were selected for their chemical properties, and finally, the sol–gel Carbowax^®^ coated FPSE membrane was chosen as the optimum of the three. A factorial experimental design, which minimizes the number of experiments and, consequently, the use of organic solvents and other pollutant materials, was used to evaluate the effects of the variables that are involved in the FPSE process and to choose the best combination of them. The results reveal that this method is a promising alternative to traditional extraction procedures, such as SPE, because of the low quantities of solvents used, moderate extraction efficiencies achieved, and the appropriate detection limits for the determination of hormonal contamination of milk samples. Furthermore, the elimination of the pretreatment process, including protein precipitation and defatting of milk samples, is an important advantage of the optimized methodology.

The special characteristics of the milk samples, as well as the risk of simultaneously co-extracting significant amounts of matrix interferents, such as proteins or lipids, with the target analytes, make it necessary to develop extraction procedures that can overcome the problems associated with these unwanted matrix components. Therefore, a study on how the fat content in milk affects the extraction procedure was conducted, and it was observed that the higher the amount of fat, the higher the loss in the extraction efficiency. Furthermore, for the first time, the effect of lactose on the extraction step was evaluated, and it was observed that extraction efficiency was enhanced in lactose-free milk. This indicates that lactose could be considered an interferent, similar to proteins or fat, in the extraction of organic pollutants from milk.

The optimized FPSE–UHPLC–MS/MS methodology was applied to several samples of cow milk with different amounts of fat content, as well as to goat milk, and target hormones were not detected in any sample. The method had detection limits between 0.047 and 1.242 ng·mL^−1^, which is appropriate for the determination of contamination by steroid hormones in milk samples. Given its simplicity, ease of use, and other advantages, the new FPSE–UHPLC–MS/MS method could be adopted to evaluate the quality assurance of milk samples by confirming the absence of steroid hormones that showed good extraction efficiencies, such as androgens and progestogens, and thus ensure food safety and quality.

## Figures and Tables

**Table 1:** List of target hormones studied and their acronyms, chemical structures, CAS numbers, and log K_OW_ values. Data were extracted from the PubChem database.

Hormone		Chemical Structure	CAS Number	Log K_ow_
ESTROGENS	Estrone (E1)	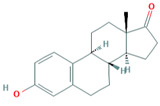	53-16-7	3.13
	17β-Estradiol (E2)	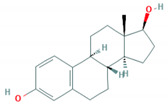	50-28-2	4.01
	17α-Ethynylestradiol (EE)	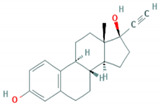	57-63-6	3.67
	Estriol (E3)	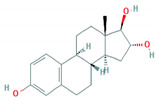	50-27-1	2.45
	Diethylstilbestrol (DES)	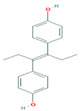	56-53-1	5.07
PROGESTOGENS	Levonorgestrel (NOR)	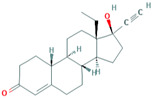	797-63-7	3.8
	Norethisterone (NORET)	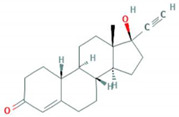	68-22-4	2.97
	Megestrol acetate (MGA)	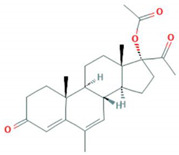	595-33-5	3.2
	Progesterone (PRO)	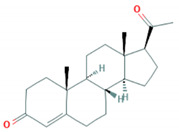	57-83-0	3.87
ANDROGENS	Testosterone (TES)	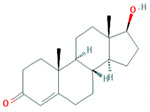	58-22-0	3.32
	Boldenone (BOL)	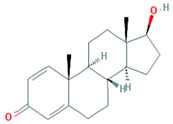	846-48-0	3.05
	Nandrolone (NAN)	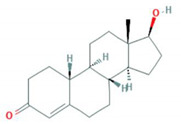	434-22-0	2.62
GLUCOCORTICOIDS	Cortisone (COR)	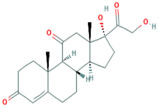	53-06-5	1.47
	Prednisone (PRD)	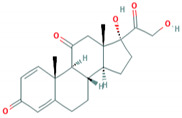	53-03-2	1.46
	Prednisolone (PRDNL)	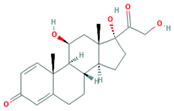	50-24-8	1.62

**Table 2:** Low and high values for the optimized variables of FPSE.

Variable	Low Value (−)	High Value (+)
Sample volume	1 mL	5 mL
Extraction time	10 min	30 min
Extractant volume	1 mL	2 mL
Elution time	5 min	10 min

**Table 3:** Values of target variables in the factorial experimental designs performed with the FPSE media evaluated.

Run Order	Sample Volume	Extraction Time	Elution Time	Extractant Volume
1	+	+	−	+
2	+	+	+	+
3	−	−	+	+
4	−	+	+	−
5	−	+	−	+
6	−	−	−	−
7	+	+	+	−
8	+	+	−	−
9	+	−	+	+
10	+	−	−	−
11	+	−	+	−
12	−	−	−	+
13	−	+	−	−
14	−	−	+	−
15	−	+	+	+
16	+	−	−	+

**Table 4:** Analytical parameters for the target steroid hormones.

Hormone	Internal Standard	Linear Regression Coefficient (r^2^)	MDL (ng·mL^−1^)	MQL (ng·mL^−1^)	Recovery ± SD (%)
E1	E1–d2	0.9936	0.27	0.91	46.47 ± 7.10
E2		0.9977	0.53	1.78	38.04 ± 8.64
EE		0.9938	1.24	4.14	28.60 ± 19.42
E3		0.9940	0.32	1.07	25.41 ± 8.42
DES		0.9923	0.20	0.68	21.89 ± 4.63
NOR	PRO–d9	0.9969	1.08	3.60	45.29 ± 8.45
NORET		0.9935	0.05	0.15	53.51 ± 9.54
MGA		0.9928	0.01	0.04	45.48 ± 4.26
PRO		0.9924	0.14	0.46	49.57 ± 6.55
TES	TES–d3	0.9940	0.21	0.69	50.09 ± 2.79
BOL		0.9934	0.07	0.24	49.03 ± 7.25
NAN		0.9936	0.24	0.81	59.01 ± 5.30
COR	PRO–d9	0.9944	0.01	0.04	22.07 ± 4.62
PRD		0.9979	0.29	0.96	23.89 ± 6.78
PRDNL		0.9919	0.05	0.16	17.91 ± 2.59

## Supplementary Materials

The following are available online at https://www.mdpi.com/2304-8158/10/2/343/s1, Figure S1: Pareto charts and normal plots obtained in the Carbowax© 20M variable optimization for target steroid hormones; Table S1: Quantification and confirmation fragment ions and their respective detection parameters for the compounds under study; Table S2: Model equations for Carbowax© 20M, PEG300, and PTHF fabric media and theoretical recoveries for target steroid hormones; Table S3: Partial correlations between the four studied variables and the 15 steroid hormones under study for Carbowax© 20M FPSE device media.
